# Supplementary material for: Deep Learning Histology for Prediction of Lymph Node Metastases and Tumor Regression after Neoadjuvant FLOT Therapy of Gastroesophageal Adenocarcinoma
Source: Cancers (Basel). 2024 Jul 3;16(13):2445. doi: 10.3390/cancers16132445 (PMC11240557; doi:10.3390/cancers16132445)
Supplement: Supplementary file 1 [file cancers-16-02445-s001.zip › cancers-3068063-supplementary.pdf]

Table S1. Final hyperparameters after optimization for both models, predicting lymph node status and residual vital tumor. The models were different regarding data augmentation, dropout rate, number of epochs, learning rate, loss function and balancing. Abbreviations: RVT = residual vital tumor.

hyperparameter	ypN0 vs. ypN+	RVT <50% vs. RVT >50%
augment	xyr	n
batch_size	48	48
drop_images	false	false
dropout	0.1	0
early_stop	false	false
early_stop_method	loss	loss
early_stop_patience	0	0
epochs	3	5
hidden_layer_width	500	500
hidden_layers	0	0
include_top	true	true
l1	0.0	0.0
l1_dense	0.0	0.0
l2	0.0	0.0
l2_dense	0.0	0.0
learning_rate	5e-05	1e-04
learning_rate_decay	0	0
learning_rate_decay_steps	100000	100000
loss	sparse_categorical_crossentropy	mean_squared_error
manual_early_stop_batch	null	null
manual_early_stop_epoch	null	null
model	xception	xception
normalizer	macenko	macenko
normalizer_source	null	null
optimizer	Adam	Adam
pooling	max	max
tile_px	299	299
tile_um	100	100
toplayer_epochs	0	0
trainable_layers	0	0
training_balance	category	patient
uq	false	false
validation_balance	none	none

Table S2. The architecture was identical for both models, lymph node status and residual vital tumor. However, in bold and parentheses are shown the parameters for the RVT model slightly diverging due to the different loss function.

Layer (type)	Output Shape	Param #	Connected to
tile_image (InputLayer)	[(None, 299, 299, 3)]	0	[]
xception (Functional)	(None, 2048)	20861480 **(20861480)**	['tile_image[0][0]']
post_convolution (Activation)	(None, 2048)	0	['xception[0][0]']
slide_feature_input (InputLayer)	[(None, 23)]	0	[]
dropout (Dropout)	(None, 2048)	0	['post_convolution[0][0]']
input_merge (Concatenate)	(None, 2071)	0	['slide_feature_input[0][0]', 'dropout[0][0]']
logits-0 (Dense)	(None, 2)	4144 **(2072)**	['input_merge[0][0]']
out-0 (Activation)	(None, 2)	0	['logits-0[0][0]']
Total params: 20,865,624 **(20,863,552)**<br>Trainable params: 20,811,096 **(20,809,024)**<br>Non-trainable params: 54,528 **(54,528)**			
